# Characteristics of Angiotensin I-converting enzyme 2, type II transmembrane serine protease 2 and 4 in tree shrew indicate it as a potential animal model for SARS-CoV-2 infection

**DOI:** 10.1080/21655979.2021.1940072

**Published:** 2021-07-06

**Authors:** Na Li, Wenpeng Gu, Caixia Lu, Xiaomei Sun, Pinfen Tong, Yuanyuan Han, Wenguang Wang, Jiejie Dai

**Affiliations:** The Center of Tree Shrew Germplasm Resources, Institute of Medical Biology, Chinese Academy of Medical Sciences and Peking Union Medical College, Yunnan Key Laboratory of Vaccine Research and Development on Severe Infectious Diseases, Yunnan Innovation Team of Standardization and Application Research in Tupaia Belangeri Chinensis, Kunming, China

**Keywords:** Tree shrew, ACE2, TMPRSS2, TMPRSS4, tissue expression

## Abstract

Angiotensin I-converting enzyme 2 (ACE2), type II transmembrane serine protease 2 and 4 (TMPRSS2 and TMPRSS4) are important receptors for SARS-CoV-2 infection. In this study, the full-length tree shrew*ACE2* gene was cloned and sequenced, and its biological information was analyzed. The expression levels of ACE2, TMPRSS2 and TMPRSS4 in various tissues or organs of the tree shrew were detected. The results showed that the full-length *ACE2* gene in tree shrews was 2,786 bp, and its CDS was 2,418 bp, encoding 805 amino acids. Phylogenetic analysis based on the CDS of *ACE2* revealed that tree shrews were more similar to rabbits (85.93%) and humans (85.47%) but far from mice (82.81%) and rats (82.58%). In silico analysis according to the binding site of SARS-CoV-2 with the ACE2 receptor of different species predicted that tree shrews had potential SARS-CoV-2 infection possibility, which was similar to that of rabbits, cats and dogs but significantly higher than that of mice and rats. In addition, various tissues or organs of tree shrews expressed ACE2, TMPRSS2 and TMPRSS4. Among them, the kidney most highly expressed ACE2, followed by the lung and liver. The esophagus, lung, liver, intestine and kidney had relatively high expression levels of TMPRSS2 and TMPRSS4. In general, we reported for the first time the expression of ACE2, TMPRSS2 and TMPRSS4 in various tissues or organs in tree shrews. Our results revealed that tree shrews could be used as a potential animal model to study the mechanism underlying SARS-CoV-2 infection.

## Graphical abstract

**Figure:**
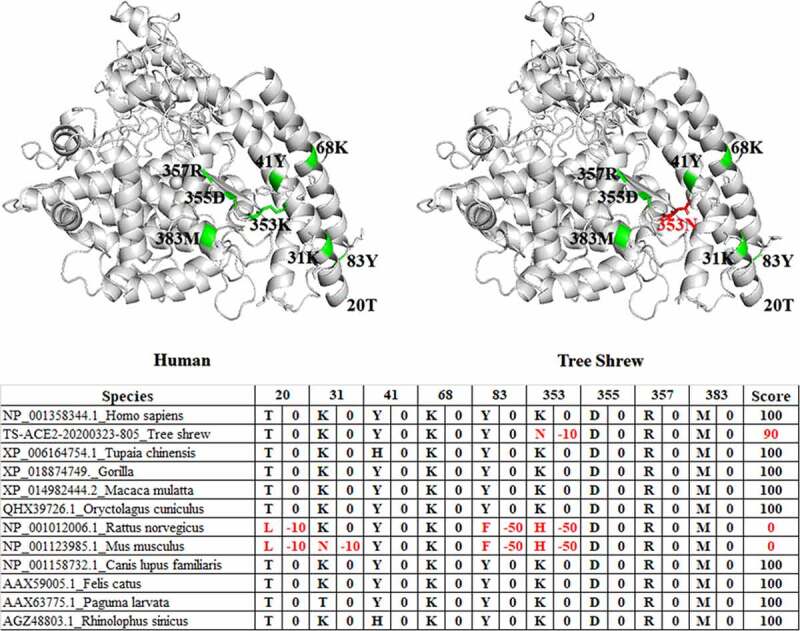

## Introduction

In 2020, the new coronavirus pneumonia virus (SARS-CoV-2) caused large-scale outbreaks of coronavirus disease 2019 (COVID-19) in various parts of the world and led to the global pandemic. As of June 2020, the World Health Organization reported more than 130 million cases and 500,000 deaths caused by SARS-CoV-2 infection, thereby provoking a serious threat to human health and social development. A recent study found [1] that SARS-CoV-2 was highly homologous to a bat coronavirus, which could be attributed to the coronavirus genus, and both viruses had the same cellular receptor, angiotensin I-converting enzyme 2 (ACE2).

ACE2 is a type I transmembrane glycoprotein with a single extracellular catalytic domain and belongs to the angiotensin-converting enzyme family. The amino terminal zinc metalopeptidase domain has 41.8% homology with angiotensin-converting enzyme and can be used as a carboxypeptidase, acting on angiotensin (Ang), bradykinin, neurotensin, kinetensin and other substrates [2]. The carboxyl-terminal domain has 48% sequence identity with the noncatalytic protein collectrin and plays a role in regulating amino acid transport [3]. ACE2 has many important pathophysiological roles in humans and animals.

The attachment of SARS-CoV-2 to the target cell is initiated by interactions between the spike (S) glycoprotein and ACE2. Following receptor engagement, the SARS-CoV-2 S protein is processed by plasma membrane-associated type II transmembrane serine protease 2 (TMPRSS2) and TMPRSS4 [4]. Therefore, ACE2, TMPRSS2 and TMPRSS4 are very important for SARS-CoV-2 infection and are the predominant target receptors for many human viruses.

The tree shrew (*Tupaia belangeri chinensis*) is a small mammal that looks like a squirrel. It belongs to the order Scandentia and has a close relationship with primates. It can be used for biomedical studies of various human diseases [5–7]. In recent years, several virus infection models, including influenza B [8], Zika virus [9], hepatitis C virus [10], and avian H5N1/H9N2 influenza viruses [11,12], have been investigated by using tree shrews. Other human diseases, such as retinal vein pulsation and glaucoma of the visual system [13–15], myocardial ischemia and atherosclerosis of the cardiovascular system [16,17], nonalcoholic fatty liver [18], diabetic nephropathy [19] and obesity [20] of metabolism, cancers [21,22], Alzheimer’s disease [23] and depression [24], all used tree shrews as disease models for their studies. However, the characteristics of ACE2, TMPRSS2 and TMPRSS4 in tree shrews have not been reported in most tissues or organs, and organs are more at risk for SARS-CoV-2 infection. Rhesus monkeys are currently the best animal model of SARS-CoV-2, but they are expensive to study, have a long study period and require rigorous conditions. In addition, what other laboratory animals are suitable as a SARS-CoV-2 research animal model? Compared with primates, tree shrews have been widely used in biomedical studies because of their advantages, such as small size, low price and low research cost. Although many human disease models established might be related to ACE2, TMPRSS2 and TMPRSS4, there has been no systemic study on ACE2, TMPRSS2 and TMPRSS4 in tree shrews.

In addition, at least two studies have reported SARS-CoV-2 infection in tree shrews. Results of one study indicated that tree shrews could be infected by SARS-CoV-2 and have the potential to be used as an animal model for SARS-CoV-2 infection [25]. Another study revealed that tree shrews were less susceptible to SARS-CoV-2 infection than other reported animal models but could be a potential intermediate host of SARS-CoV-2 as they are asymptomatic carriers [26]. Therefore, it is very important to analyze the receptors for virus infection of animal hosts to further evaluate the usage of the tree shrew as an animal model for SARS-CoV-2 or other diseases. In this study, we cloned the ACE2 gene of tree shrews, carried out bioinformatics analysis, detected the expression of ACE2, TMPRSS2 and TMPRSS4 in various tissues of tree shrews to assess the risk of tree shrew infections with SARS-CoV-2, and established ACE2-, TMPRSS2- and TMPRSS4-related disease models in tree shrews.

## Materials and methods

### Animal sources

Tree shrews were in the closed population and selected from The Center of Tree Shrew Germplasm Resources, Institute of Medical Biology, and Chinese Academy of Medical Sciences. Animals were divided into 3 groups according to age: group one (0–3 months), group two (3 years), and group three (>6 years old). There were 2 animals in each group (one male and one female).

The animal’s different tissues were collected following anesthesia and euthanasia method: tree shrews were cervical dislocated 5 min following an intraperitoneal injection of 2% pentobarbital sodium (0.2 ml/100 g, Sigma-Aldrich, USA). The sample collections were performed in accordance with relevant guidelines and regulations approved by the Ethical Committee of Institute of Medical Biology, Chinese Academy of Medical Sciences and Peking Union Medical College. All experimental procedures were approved by the Ethics Review Committee (Institutional Review Board [IRB]) of Institute of Medical Biology, Chinese Academy of Medical Sciences and Peking Union Medical College. All applicable institutional and/or national guidelines for the care and use of animals were followed.

### Primers design and gene amplification

The primers were designed according to the predicted sequence of the tree shrew ACE2 gene (accession number: XM_006164692.3) published on NCBI. Tree shrew small intestine tissue was selected as the sample, and total RNA was extracted from the tissue according to the RNAiso Plus reagent instructions (Takara, Japan). The reverse transcription reaction was performed according to the instructions of the PrimeScript II 1st Strand cDNA Synthesis Kit (Takara, Japan). Using the reverse transcribed cDNA as a template, PCR was performed based on the instructions of Premix Taq (La Taq Version 2.0, Takara, Japan), and the amplified products were detected by 1% agarose gel electrophoresis.

The target fragment was expected to be 2,255 bp in length, and after PCR amplification, the products were sent for sequencing (Takara, Japan). According to the sequencing results, 5ʹ-end and 3ʹ-end RACE experiments were performed. After sequencing verification, full-length splicing was completed (2,786 bp), and final verification was performed by using verification primers. All the primers used in this study are shown in Table 1.

**Table 1.** The primers used for Real-Time qPCR in this study

Primers	Sequences	Length	Purpose
ACE2-F	5ʹ-GCCTYGTTGCTGTAACTGCTGCTC-3’	2,255 bp	PCR
ACE2-R	5ʹ-CATCASTGTTTTGGAATCCTGGAT-3’		
ACE2-YZF1	5ʹ-ATCTTGGCATAGAGGGAAAGATGG-3’	2,786bp	PCR
ACE2-YZR1	5ʹ-CATTTCTGATATCCCTGAATAGCC-3’		
ACE2-F1	5ʹ- ACTGGATGCCTCCCTGCTCA −3’	173bp	RT-qPCR
ACE2-R1	5ʹ- GTCCCAAGCTGTAGGGTGAC −3’		
GAPDH-F	5ʹ- GCGAGATCCCGCCAACATCA-3’	150bp	RT-qPCR
GAPDH-R	5ʹ- GTCCCTCCACGATGCCGAAG −3’		
TMPRSS2-F	5ʹ- CATGCGAGGACATGGGCTAT-3’	230bp	RT-qPCR
TMPRSS2-R	5ʹ- TGATCCACCCACAATCCTGC-3’		
TMPRSS4-F	5ʹ- CCCAATGAGACAAGTGCAGC-3’	238bp	RT-qPCR
TMPRSS4-R	5ʹ- GTTGGTCACTGTCGTGGTCT-3’		

Note: ACE2: Angiotensin I-converting enzyme 2; TMPRSS2: type II transmembrane serine protease 2; TMPRSS4: type II transmembrane serine protease 4.

### Bioinformatics analysis

Phylogenetic analysis was performed with NCBI’s online BLAST and BioEdit programs, and Mega 7.0 software was used to build a phylogenetic tree. ProtParam was used to analyze the amino acid composition, theoretical molecular mass and isoelectric point of the encoded protein; NPS@MLRC predicted the secondary structure of the protein; SignlP predicted the protein N-terminal signal peptide; TMHMM and OCTOPUS were used to analyze the transmembrane distribution of the protein; ProtScale predicted the hydrophilicity and hydrophobicity of the protein; NetNGlyc software analyzed the glycosylation sites; the k-NN program predicted the protein subcellular localization; SMART, CDD tool and InterProScan were used to analyze the possible protein domains; and SWISS-MODEL predicted the tertiary structure of the protein. According to previous studies on the binding site of coronaviruses and the human ACE2 protein, the differences in the tree shrew ACE2 protein were analyzed, and the risk of infection was predicted [27,28].

### Expression of ACE2 in various tree shrew tissues or organs

Total RNA of various tree shrew tissues or organs, including heart, liver, spleen, lung, kidney, brain, bone marrow, muscle and skin, was extracted with the RNAiso Plus reagent, and GAPDH was used as an internal control. Real-time quantitative PCR, according to the instructions of the One Step TB Green PrimeScript PLUS RT-PCR Kit, was used to analyze the relative mRNA expression of the *ACE2, TMPRSS2* and *TMPRSS4* genes. The 2^−ΔΔCt^ method was used to calculate the relative mRNA expression results. The primers used for the gene expression study are shown in Table 1.

Western blot analysis was conducted to examine the expression of ACE2, TMPRSS2 and TMPRSS4 in the tissues mentioned above according to a previous study. Briefly, RIPA lysis buffer (Beyotime, Shanghai) was used to extract the total proteins from the tree shrew tissues, and SDS-PAGE was conducted to separate the proteins. After that, the proteins were transferred onto a PVDF membrane and blocked with 5% skim milk; then, primary antibodies against β-actin (1:2000, Affinity Biosciences, USA) and ACE2, TMPRSS2 and TMPRSS4 (1:2000, Thermo Fisher, USA) were incubated with the PVDF membranes at 4°C overnight. Then, the membranes were incubated for 1 hour with secondary antibodies (1:5000, Affinity Biosciences, USA). The ECL system (Bio-Rad, USA) was employed to visualize the protein bands, which were further quantified by ImageJ software and normalized to β-actin.

### Statistical analysis

Statistical analyses were performed using SPSS (version 20.0, IBM, USA). The Kolmogorov-Smirnov test, t-test, ANOVA, or Kruskal-Wallis H test was used as appropriate. A P value <0.05 was considered to indicate statistical significance.

## Results

It is very important to analyze the receptors for virus infection of animal hosts to further evaluate the usage of the tree shrew as an animal model for SARS-CoV-2 or other diseases. In this study, we cloned the ACE2 gene of tree shrews, carried out bioinformatics analysis, detected the expression of ACE2, TMPRSS2 and TMPRSS4 in various tissues of tree shrews to assess the risk of tree shrew infections with SARS-CoV-2, and established ACE2-, TMPRSS2- and TMPRSS4-related disease models in tree shrews. In general, we reported for the first time the expression of ACE2, TMPRSS2 and TMPRSS4 in various tissues or organs in tree shrews. Our results revealed that tree shrews could be used as a potential animal model to study the mechanism underlying SARS-CoV-2 infection.

### Phylogenetic analysis of tree shrew ACE2

**Figure 1.**
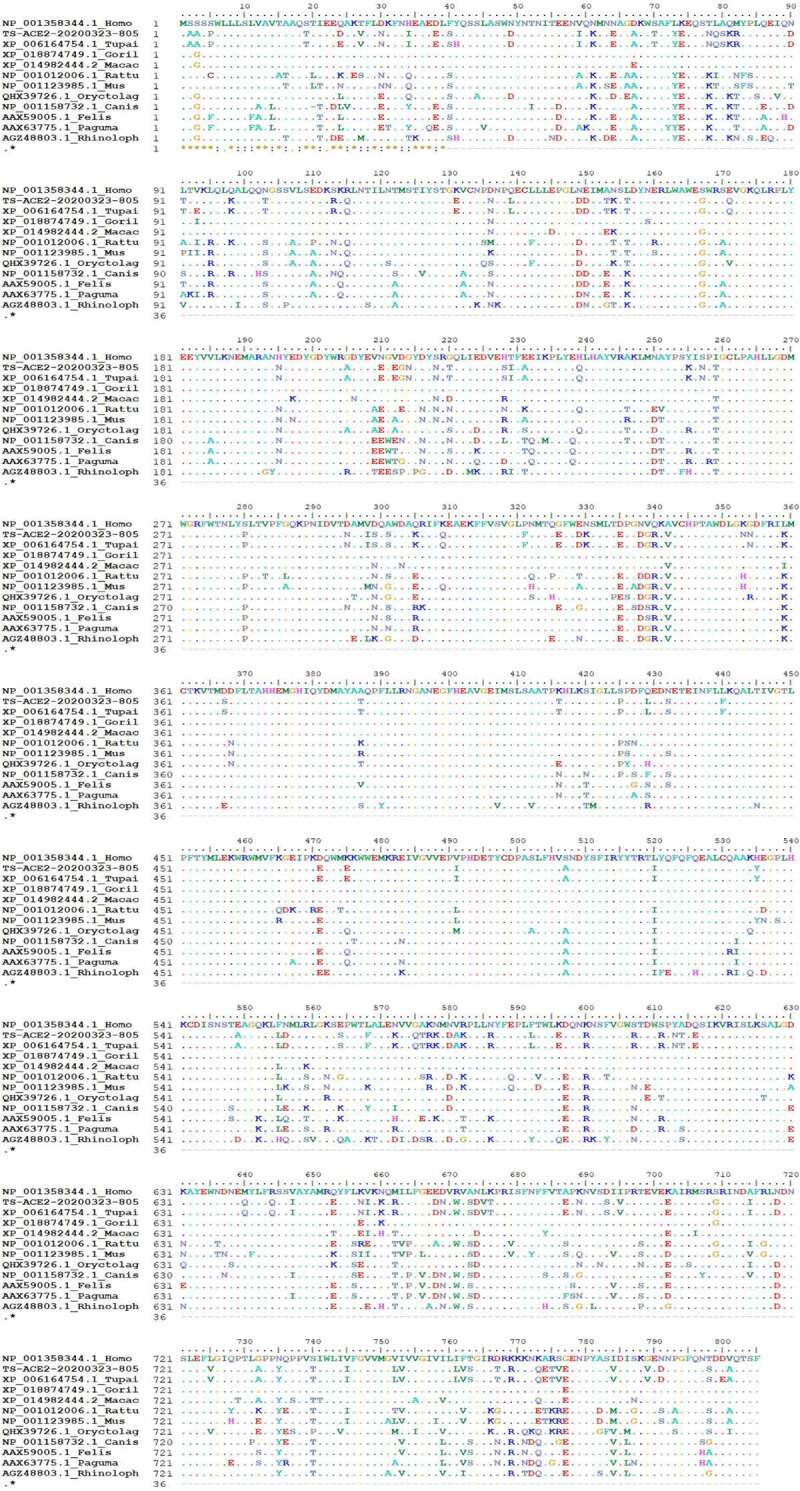
Comparison of the ACE2 amino-acid sequences of tree shrew with other species

The effective sequence length of the tree shrew ACE2 gene was 2,255 bp, the 5ʹ RACE was 112 bp, and the 3ʹ RACE was 419 bp. After verification, the full-length sequence was 2,786 bp. The sequence was submitted to GenBank with accession number MT253740, and its CDS was 2,418 bp in length and showed 99.38% identity to the predicted sequence of the tree shrew ACE2 gene and encoded 805 amino acids; the different sites were D24E, Y41H, V93E, N353K and D800E (Figure 1). The CDS and protein sequence of the tree shrew ACE2 gene (TS-ACE2-20,200,323-805) were compared with the human, gorilla, macaque, rat, mouse, rabbit, dog, cat, civet and Chinese chrysanthemum bat ACE2 genes on NCBI. The CDS of tree shrew ACE2 were more similar to that of rabbits (85.93%), gorillas (85.56%), *Macaca mulatta* (85.53%) and humans (85.47%) but were less similar to that of mice (82.81%) and rats (82.58%); the protein sequence of tree shrew ACE2 was more similar to that of cats (81.74%), gorillas (81.36%), *Macaca mulatta* (81.36%) and humans (81.11%) but were less similar to that *Paguma larvata* (79.88%) and Chinese chrysanthemums bats (77.58%), as shown in Figure 1 and Table 2.

**Table 2.** Homologous matching rates of tree shrews CDS and protein sequences with other species

Species	CDS ID	Identity	Protein ID	Identity
Tupaia belangeri chinensis	GenBank MT253740	1	TS-ACE2-20200323-805	1
Tupaia chinensis	XM_006164692.3	99.63%	XP_006164754.1	99.38%
Homo sapiens	NM_001371415.1	85.47%	NP_001358344.1	81.11%
Gorilla	XM_019019204.1	85.56%	XP_018874749.1	81.36%
Macaca mulatta	XM_015126958.2	85.53%	XP_014982444.2	81.36%
Rattus norvegicus	NM_001012006.1	82.58%	NP_001012006.1	80.23%
Mus musculus	NM_001130513.1	82.81%	NP_001123985.1	79.97%
Oryctolagus cuniculus	MN099288.1	85.93%	QHX39726.1	80.98%
Canis lupus familiaris	NM_001165260.1	85.07%	NP_001158732.1	80.79%
Felis catus	AY957464.1	84.69%	AAX59005.1	81.74%
Paguma larvata	AY881174.1	83.28%	AAX63775.1	79.88%
Rhinolophus sinicus	KC881004.1	83.69%	AGZ48803.1	77.58%

**Figure 2.**
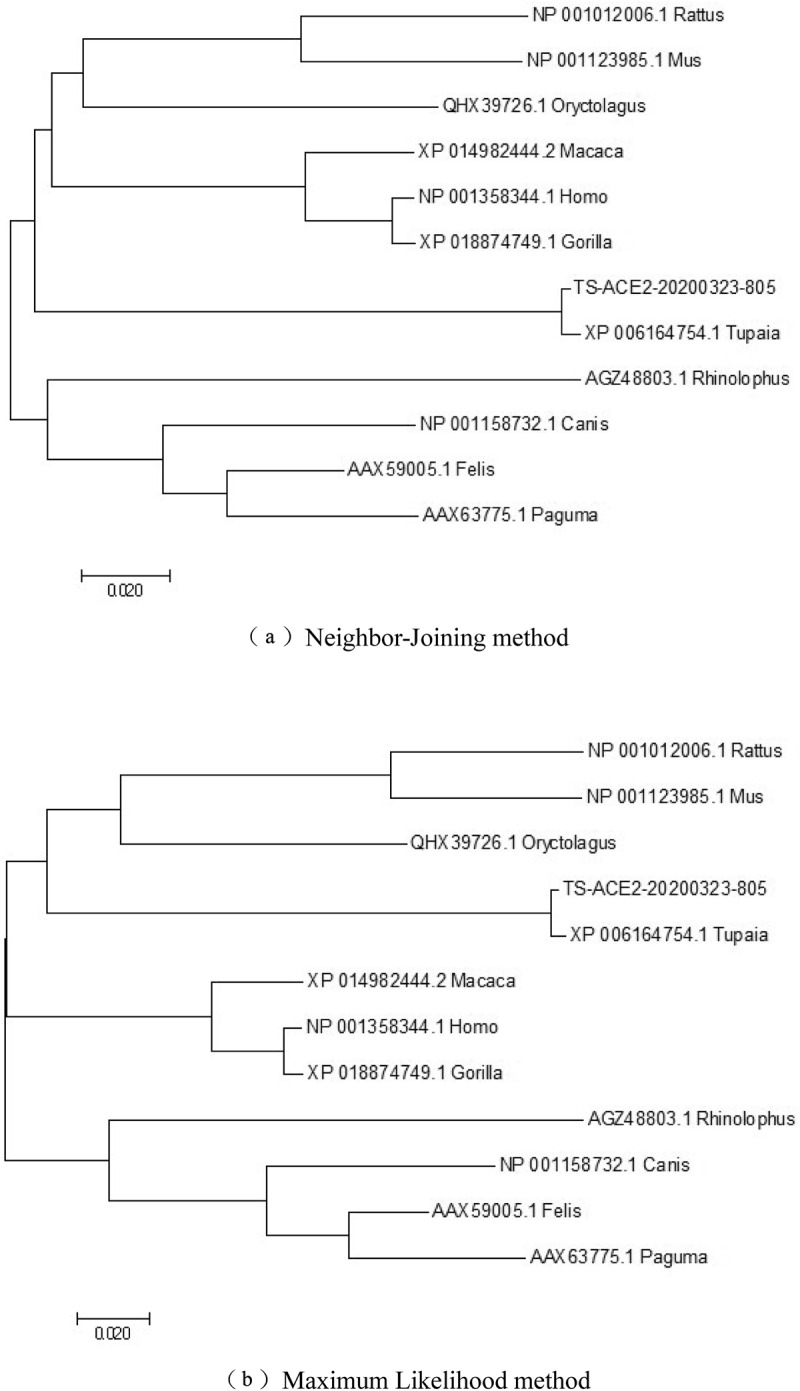
Phylogenetic tree of ACE2 protein sequence in different species

Phylogenetic tree analysis based on the neighborhood-joining method revealed that the tree shrew ACE2 gene clustered with that of humans, gorillas and *Macaca mulatta* (Figure 2. A); the maximum likelihood tree indicated that tree shrews clustered with *Oryctolagus cuniculus*, as shown in Figure 2b. Therefore, these data implied that the tree shrew ACE2 shares a common origin with nonhuman primates and rabbits.

### Bioinformatics of tree shrew ACE2

The tree shrew ACE2 protein molecular weight was estimated to be 93,003.50 Da; there are 20 kinds of amino acids in the tree shrew ACE2 protein, with high proportions with leucine (8.9%), glutamic acid (8.6%), threonine (6.2%), alanine (6.2%), valine (6.1%), aspartic acid (5.8%) and isoleucine (5.6%). The isoelectric point was 5.00, and the instability coefficient was 43.94, with a fat coefficient of 80.55; and the total average hydrophilicity (GRAVY) was −0.399. The signal peptide was present at amino acids 1–17, the signal peptide cleavage site (TAA-QT) was between amino acids 17 and 18 and there was a transmembrane structure at amino acids 740–762. It was predicted that the tree shrew ACE2 protein had N-glycosylation sites at amino acids 53, 78, 218, 257, 322, and 690. In total, 22.2% of the amino acids were distributed in the cell membrane, 44.4% were distributed in the endoplasmic reticulum, and 33.3% were distributed in the Golgi apparatus. The secondary structure of the tree shrew ACE2 protein showed that the ACE2 protein is 49.57% α-helical, 7.83% β-sheet, and 42.61% random coil.

**Figure 3.**
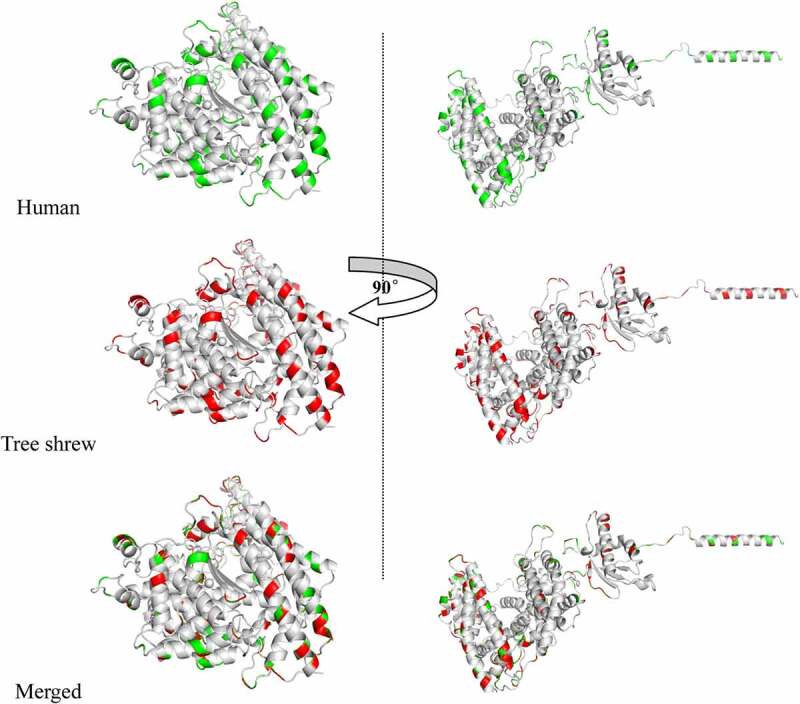
Tertiary structure prediction of ACE2 protein for tree shrews and human

**Figure 4.**
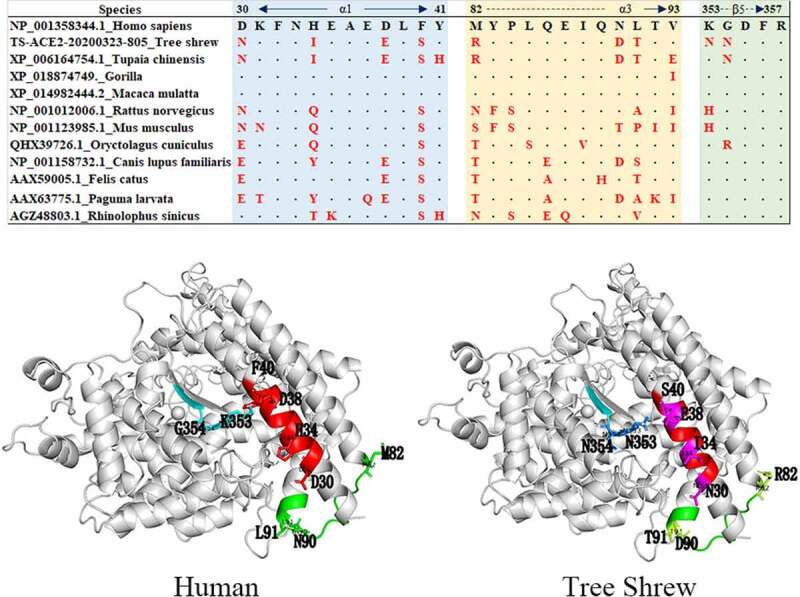
Differences of the key regions of ACE2 combined with SARS-CoV between tree shrew and human

**Figure 5.**
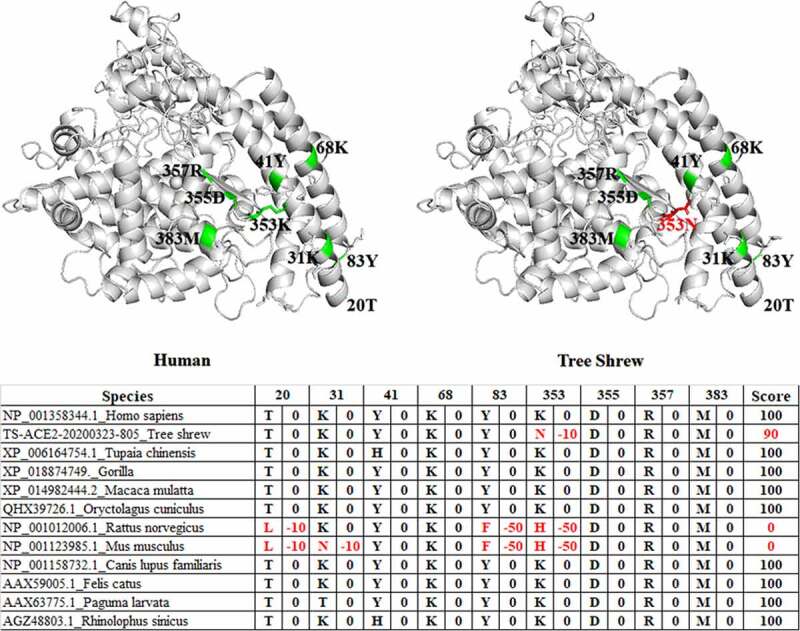
Differences of the key regions of ACE2 combined with SARS-CoV2 between tree shrew and human

SWISS-MODEL for predicting the protein tertiary structure showed that the tertiary structure of tree shrew and human ACE2 protein were basically similar, and the different sites are distinguished in red and green in Figure 3. According to previous studies, the α1 ridge region, loop and α3 region, loop and β5 region of the human ACE2 protein are the key binding regions of SARS-CoV. We evaluated the receptor utilization capacity of ACE2 among different species and ranked them to predict their infectious risk. There were 9 differences in amino acid binding sites in the tree shrew protein compared with the human protein, and these differences included D30N, H34I, D38E, F40S, M82R, N90D, L91T, K353N and G354N (Figure 4). A recent study based on phylogenetic clusters and sequence alignments of SARS-CoV-2 utilization of human ACE2 established a method to evaluate the possibility of SARS-CoV-2 infection in different species. The evaluation method described [28] that if the key positions were T20+, K/T31+, Y/H41+, K68+, Y83+, K353+, D355+, R357+, and M383, then the score was 100 points; if the mutations were K31D, Y41A, Y83F, K353H/A/D, D355A, and R357A, then each replacement subtracted 50 points from the score; if other mutations happened, then each of these mutations subtracted 10 points from the score. A final high score meant that there was a high risk of SARS-CoV-2 infection. According to this evaluation method, we conducted a risk assessment of ACE2 among different species, and the results of the risk assessment for infection by SARS-CoV-2 showed that the tree shrew had a score of 90, which is a higher risk of infection score than that of the mouse (Figure 5). Based on these results, we proposed that the tree shrew might be a potentially suitable animal model to develop an ACE2-related disease model.

### ACE2, TMPRSS2 and TMPRSS4 are expressed in various tree shrew tissues

**Figure 6.**
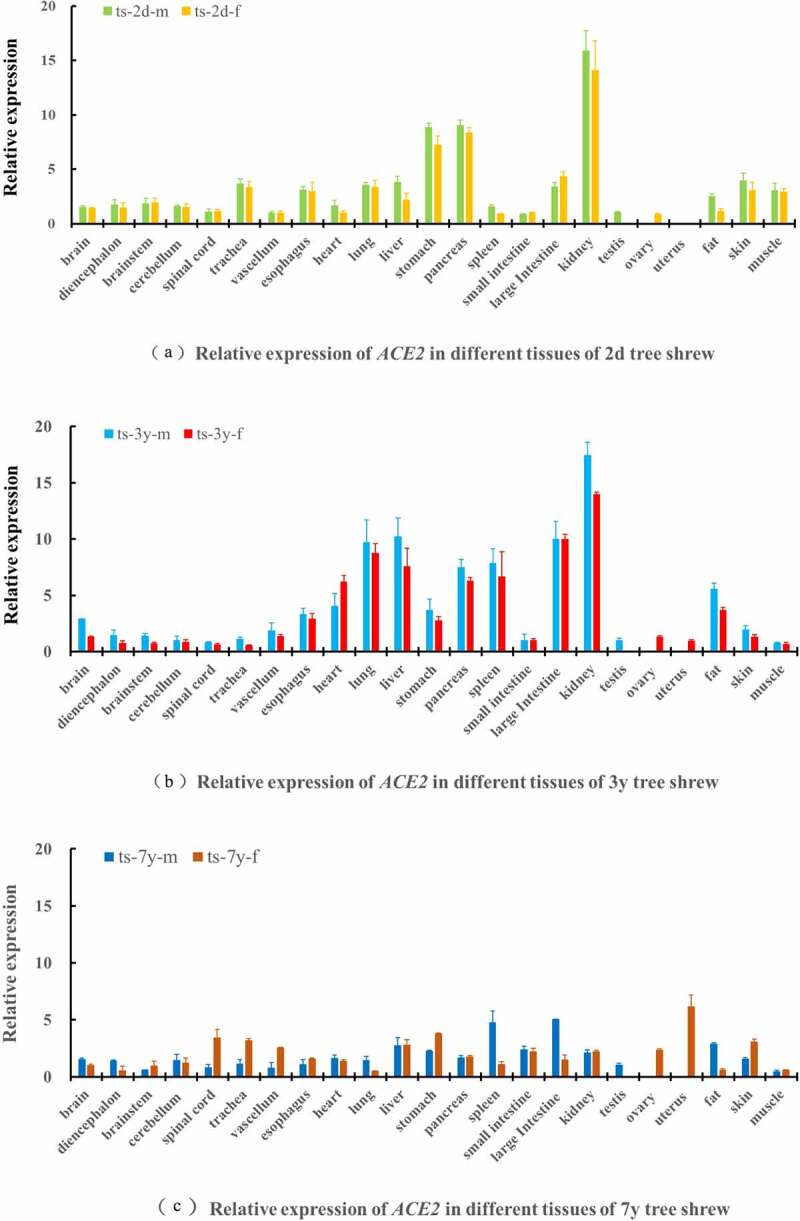
Expression of *ACE2* gene in different tissues of tree shrew

**Figure 7.**
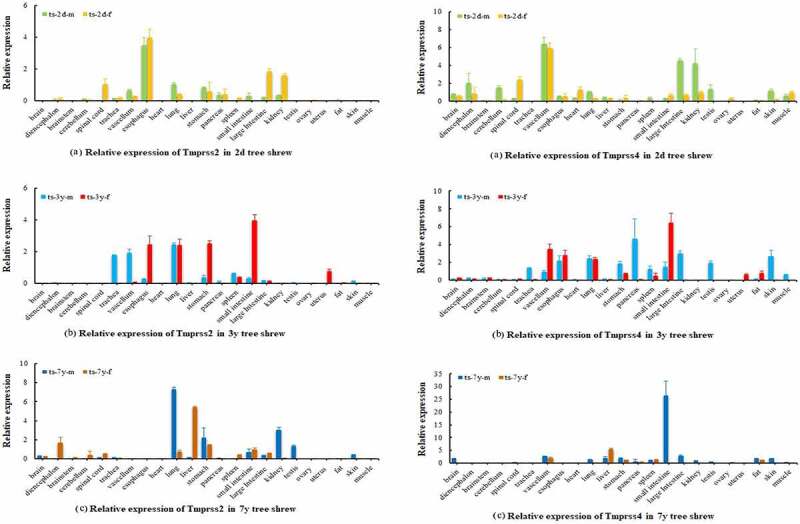
Expression of *TMPRSS2* and *TMPRSS4* gene in different tissues of tree shrew

**Figure 8.**
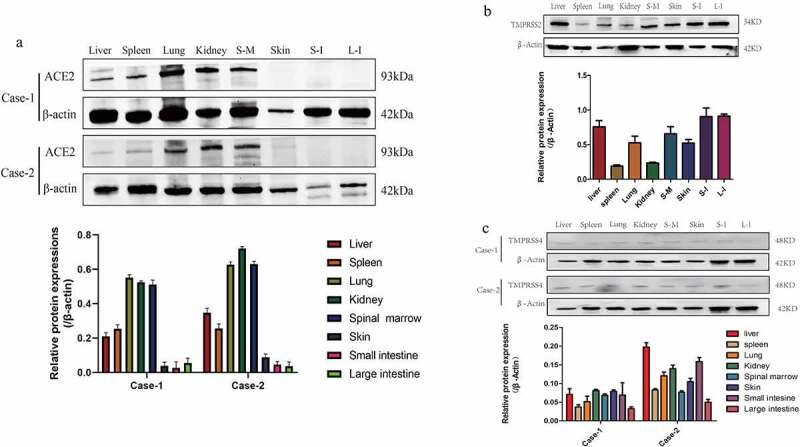
Western Blot expression levels of the Tree shrew ACE2, TMPRSS2 and TMPRSS4 proteins in different tissues. (a) WB result of ACE2 in different tissues of tree shrew; (b) WB result of TMPRSS2 in different tissues of tree shrew; (c) WB result of TMPRSS4 in different tissues of tree shrew. Each experiment had three repetitions

The mRNA expression results showed that the *ACE2, TMPRSS2* and *TMPRSS4* genes were expressed in various tree shrew tissues at different ages. Relatively high expression levels of *ACE2* in the kidney, stomach and pancreas were found in group one (2 days); the kidney, lung, liver, large intestine, pancreas, and spleen had relatively high expression levels in group two (3 years); and the expression levels of ACE2 in the uterus, spleen, and large intestine were higher in group three (7 years), as shown in Figure 6. *TMPRSS2* and *TMPRSS4* had relatively high expression levels in the esophagus, large intestine and kidney in group one (2 days); *TMPRSS2* and *TMPRSS4* had higher expression levels in the esophagus, lung, stomach, small intestine and uterus in group two (3 years); and higher expression levels of *TMPRSS2* and *TMPRSS4* were found in the lung, stomach, kidney and testis in group three (7 years), as shown in Figure 7. In general, higher relative expression levels of the *ACE2, TMPRSS2* and *TMPRSS4* genes were found in the young age groups of tree shrews, and the old age group showed lower expression levels of these genes. Western blot results indicated similar mRNA expression levels among tree shrew tissues or organs, and the kidney, lung, liver, spleen and spinal marrow had relatively high expression levels of the tree shrew ACE2, TMPRSS2 and TMPRSS4 proteins, as shown in Figure 8. We speculated that the tree shrew kidney, lung, esophagus, and large intestine might be suitable for developing cell models for SARS-CoV-2 infection.

Ts-2d-m: tree shrew aged two days for male; ts-2d-f: tree shrew aged two days for female; ts-3y-m: tree shrew aged three years for male; ts-3y-f: tree shrew aged three years for female; ts-7y-m: tree shrew aged seven years for male; ts-7y-f: tree shrew aged seven years for female.

Ts-2d-m: tree shrew aged two days for male; ts-2d-f: tree shrew aged two days for female; ts-3y-m: tree shrew aged three years for male; ts-3y-f: tree shrew aged three years for female; ts-7y-m: tree shrew aged seven years for male; ts-7y-f: tree shrew aged seven years for female.

## Discussion

As the most representative carboxypeptidase, ACE2 regulates the metabolism of angiotensin. Early studies found that it can efficiently hydrolyze angiotensin II to Ang (1–7), regulate the renin-angiotensin system (RAS) and protect the cardiovascular system by relaxing blood vessels and lowering blood pressure [29]. Subsequent studies also confirmed that ACE2 can reduce myocardial damage caused by Ang II and reverse ventricular remodeling [30]. ACE2 also increases the activity of Ang (1–7) in the kidney, inhibiting the phosphorylation of Ang II-activated mitogen-activated protein kinase (MAPK) in renal tubular cells and ultimately protecting the kidneys through antihyperplasia and diuresis activities, promoting urinary sodium excretion and reducing oxygen consumption.

ACE2 has anti-inflammatory, antiapoptotic, and antifibrotic effects on lung diseases [31]. It can reduce tissue autophagy and inflammation in acute lung injury (ALI) through the AMPK/mTOR pathway [32]. ACE2 also plays roles in many metabolic-related diseases. It can improve the metabolism of lipids and glucose in nonalcoholic fatty liver disease by combating endoplasmic reticulum stress or regulating skeletal muscle fat by improving endoplasmic reticulum and mitochondrial functions [33,34]. A recent study also showed that oral administration of lactobacillus-expressed human ACE2 in mice can improve diabetic retinopathy [35]. ACE2 is associated with various neurological diseases, such as cognition, memory, anxiety and depression [36]. Activation of ACE2 can prevent cognitive decline and reduce amyloid pathological changes [37]. The human body can use the ACE2-Ang (1-7)-MasR pathway to exert anti-anxiety and anti-depression effects [38].

In addition to its multiple functions in physiology and pathology, ACE2 is also a universal receptor for several coronaviruses. When SARS broke out in 2003, ACE2 was identified as a functional receptor for SARS-CoV [39]. A study in 2013 showed that the SARS-like coronavirus isolated from the Chinese horseshoe bat from Yunnan used ACE2 of humans, civets, and Chinese horseshoe bats as its functional receptors [40]. ACE2 is an important but ubiquitous carboxypeptidase. It is relatively conserved among different species of this protein. In our study, the homology of the *ACE2* CDS between tree shrew and human genes was 85.47%, and the protein sequence homology was 81.11%; both the homology of the *ACE2* CDS and protein sequence are higher than that between tree shrews and rats or mice.

Previous studies analyzed ACE2 from different species; the results indicated that human and nonhuman primates had identical sequences and residues in certain regions, showing that ACE2 from nonhuman primates might recognize SARS-CoV-2 and mediate its infection. They considered that nonhuman primates might be susceptible to SARS-CoV-2 and serve as animal models for medical research. However, their results also revealed that significant changes were found in mouse or rat ACE2 compared with human ACE2, assuming that rodents were not the susceptible host for SARS-CoV-2 infection [41,42].

The protein structure prediction results showed that the peptidase_M2 domain of the angiotensin-converting enzyme superfamily existed at the amino-terminus of tree shrew ACE2, the typical HEMGH zinc binding motif existed at positions 374–378, and the renal amino acid transport protein existed at the carboxy-terminus. All these data revealed that the tree shrew ACE2 protein structure was completely consistent with the human ACE2 protein structure. It was predicted that the tree shrew ACE2 protein 1–17 had a signal peptide site and multiple N-glycosylation sites. The subcellular localization was mainly the endoplasmic reticulum and Golgi apparatus, followed by the cell membrane. These characteristics were exactly the same as the functions of the carboxypeptidase. Comprehensive bioinformatics analysis results showed that the structure and physical and chemical properties of ACE2 in tree shrews were similar to those in humans, which indicated that it could play a role in the replication of human-related diseases.

In addition, recent study [4] on the expression of two mucosa-specific serine proteases, TMPRSS2 and TMPRSS4, indicated that these two proteins could facilitate SARS-CoV-2 spike protein fusogenic activity and promote virus entry into host cells. TMPRSS2 and TMPRSS4 also increased SARS-CoV-2 infectivity, at least in gut epithelial cells. ACE2, TMPRSS2 and TMPRSS4 were highly expressed in the gastrointestinal tract, particularly by intestinal epithelial cells, the predominant target cells for many human enteric viruses. Notable intestinal symptoms, including abdominal pain and diarrhea, have been observed in 20 to 50% of COVID-19 patients. A cohort-based study revealed that 61% of patients exhibited gut symptoms. All these results demonstrated that TMPRSS2 and TMPRSS4 played important roles in the SARS-CoV-2 infection process. In this study, we also found that TMPRSS2 and TMPRSS4 were expressed in different tissues or organs of tree shrews, especially in the small intestine, large intestine and lung, which suggested that tree shrews could be infected by SARS-CoV-2.

At present, two studies have been conducted on SARS-CoV-2 infection by using tree shrews. Xu et al. [25] found that Chinese tree shrews could be infected by SARS-CoV-2. The X-ray radiographs indicated lung infiltrates in most infected animals in their study, and virus was detected in lung tissues during the experimental process. Histopathological analysis showed thickened alveolar septa and interstitial hemorrhage of lung tissues. They finally concluded that tree shrews have the potential to be used as animal models for SARS-CoV-2 infection. However, Zhao et al. [26] revealed that no clinical signs were observed in SARS-CoV-2-inoculated tree shrews during the experiment. However, low levels of viral shedding and replication in tissues occurred in all age groups of animals. They found that young tree shrews showed viral shedding at the early stage of infection, and the old group had a longer duration of viral shedding. Histopathological tests indicated mild pulmonary abnormalities in infected tree shrews. Therefore, they considered that tree shrews were less susceptible to SARS-CoV-2 infection than the reported animal models but might be a potential intermediate host of SARS-CoV-2 as asymptomatic carriers. It still seems controversial for tree shrews to be used as SARS-CoV-2 infection models. From the perspective of the host for virus infection receptors, it may give us some new clues and information.

Currently, the characteristics of ACE2, TMPRSS2 and TMPRSS4 in tree shrews have not been reported in most tissues or organs, in which organs are more at risk for SARS-CoV-2. Rhesus monkeys are currently the best animal model of SARS-CoV-2, but they are expensive to study, have a long study period and require rigorous conditions. Tree shrews have recently been widely used in biomedical research, especially in virus-related infection models [43]. Tissue expression results showed that tree shrew ACE2, TMPRSS2 and TMPRSS4 are ubiquitously distributed in the body, including the major organs of the circulatory system, the respiratory system, the digestive system, the brain and spinal cord of the central nervous system, the testes, ovaries, and uterus of the reproductive system, and skin and muscles. Among them, the relative expression of ACE2 in the kidney was high in different age groups. In addition to the kidney, the expression of ACE2 in the stomach, pancreas, skin and muscle was also higher in the juvenile group than in the large intestine, lung, liver, heart and fat in the adult group. Comparing humans, tree shrews, rats and mice, ACE2 was relatively highly expressed in the kidneys; for the digestive system, humans had the highest expression in the small intestine, but tree shrews and mice had higher expression in the large intestine. In addition, the esophagus, lung, intestine and kidney had relatively high expression levels of TMPRSS2 and TMPRSS4. The different expression of ACE2, TMPRSS2 and TMPRSS4 in different age groups of tree shrews may be due to sampling procedures, animal numbers or biological characteristics. However, these results suggest that tree shrews could be used to establish a variety of ACE2-, TMPRSS2- and TMPRSS4-related disease models and provide a new idea for the exploration of related disease mechanisms.

Similar to SARS-CoV, the host of SARS-CoV-2 was likely to be a bat according to genomic homology comparison, specifically the Chinese chrysanthemum bat from Yunnan [44,45]. The host’s ACE2 was a critical factor in evaluating the possibility of infection. A previous study found that the α1 ridge region, loop and α3 region, loop and β5 region of the human ACE2 protein were the key binding regions of SARS-CoV [27]. In our study, there were 9 differences between tree shrews and humans in these above mentioned binding sites. In addition, according to the susceptible risk scheme evaluation method, we conducted a risk assessment of ACE2 in different species. There was only a key site substitution of human K353N in tree shrews. Based on the 100-point evaluation criteria, the tree shrew score was 90, which had a relatively high risk of infection, and the score in tree shrews was lower than that of rabbits, cats and dogs but much higher than that of mice. In general, we considered that tree shrews could be used as a potential animal model for SARS-CoV-2 infection.

## Conclusions

In general, we reported for the first time the expression of ACE2, TMPRSS2 and TMPRSS4 in various tissues or organs in tree shrews. Our results revealed that tree shrews could be used as a potential animal model to study the mechanism underlying SARS-CoV-2 infection.

## Data Availability

All the involved data had been included in the present manuscript, and the original raw data could be obtained from the corresponding author upon reasonable request.
